# Awareness and Sense of Urgency in Dental Avulsion Management: Knowledge Gaps Among Dutch Teens and Students

**DOI:** 10.1111/edt.70047

**Published:** 2025-12-26

**Authors:** Hiuman Maxim Chan, Jasmine Atay, Renee Helmers, Leander Dubois

**Affiliations:** ^1^ Department of Oral and Maxillofacial Surgery, Amsterdam UMC and Academic Center for Dentistry Amsterdam (ACTA) University of Amsterdam Amsterdam the Netherlands; ^2^ Department of Oral and Maxillofacial Surgery Northwest Clinics Alkmaar the Netherlands

## Abstract

**Introduction:**

According to Dutch dental trauma guidelines (DTG), optimal outcomes for avulsed permanent teeth are achieved when replantation or professional treatment occurs within 30 to 60 min following trauma. However, studies suggest that patients in the Netherlands often seek care well beyond the critical window for effective intervention, reducing the chances of successful reimplantation. This delay may be partly due to limited public awareness of appropriate first‐aid measures for dental avulsion.

**Objective:**

This study assesses awareness and perceived urgency in managing dental avulsion among Dutch students, focusing on knowledge gaps and their variation across different educational levels. Participants included secondary school students and individuals in higher education with both medical and non‐medical backgrounds.

**Methods:**

A 16‐item questionnaire, incorporating a case‐based scenario, was designed to evaluate general awareness and urgency perception. The questionnaire was distributed in educational settings between December 2024 and February 2025 and completed by students.

**Results:**

A total of 348 responses were included. Only 49% of participants correctly identified the recommended treatment timeframe of 30 min or less. Those with a dental background scored highest overall (*p* < 0.001), while secondary school students scored lowest. Medical students showed significantly greater general knowledge of avulsion management (“awareness”) than secondary school students (*p* < 0.05); however, they did not differ considerably in recognizing the urgency of treatment or in taking correct immediate actions (“sense of urgency”). Notably, only 17% of participants answered all six key management questions correctly according to national guidelines, highlighting substantial knowledge gaps, even among higher‐education groups.

**Conclusion:**

The findings of this study indicate significant knowledge deficits regarding the emergency management of dental avulsion across all educational levels. These results highlight the urgent need for targeted educational strategies to enhance first‐aid awareness, particularly among adolescents and medical students, with an emphasis on timely and appropriate response.

## 1 Introduction

Time is a crucial factor in managing dental avulsions. The maximum allowable dry time for replantation is limited to 15 min, after which periodontal ligament (PDL) viability begins to decline significantly [1]. According to both national (DTG) and international (IADT) guidelines, an avulsed tooth should ideally be treated within 30–60 min after trauma—provided the tooth has been stored in an appropriate solution to maximize its chance of long‐term survival. Beyond this critical window, the risk of complications, such as external root resorption and ankylosis, increases significantly [2, 3, 4]. For teeth with complete root development, the risk of ankylosis can rise to 86.4% after 900 days with a dry time of more than 60 min before replantation [5].

Despite these guidelines, recent Dutch data indicate that patients typically contact a healthcare provider approximately 79 min after experiencing a dental avulsion [6]. This represents a significant delay that may negatively affect the tooth's prognosis [2]. Although the underlying causes of this delay are not fully understood, inadequate public knowledge regarding the emergency management of avulsed teeth is likely a contributing factor. Previous studies across various countries have identified knowledge gaps among different target groups, such as sport coaches, parents, and dental students [7, 8, 9, 10, 11].

Several groups are particularly vulnerable to traumatic dental injuries (TDIs), especially children and adolescents aged 7–18 years, nearly half of whom sustain TDIs during recreational activities such as sports [12]. This highlights the importance of promoting awareness and first aid competence among youth, who are directly exposed to these risks in both organized sports contexts and recreational activities [13]. Given their increased vulnerability, targeted strategies to assess and enhance knowledge about dental avulsion management may yield meaningful improvements in patient outcomes.

This study examines the awareness among Dutch adolescents and students regarding the emergency management of dental avulsions, with a specific focus on the sense of urgency to seek professional care. The study population includes secondary school students and individuals enrolled in higher education, covering both medical and non‐medical disciplines. This study aims to inform the development of focused educational interventions that can improve first‐aid responses and long‐term outcomes in cases of avulsion by identifying the knowledge gaps in these groups.

## 2 Materials and Methods

### 2.1 Study Design and Population

This cross‐sectional, observational survey study was conducted between December 2024 and February 2025. Secondary school students were recruited from three different levels of education: VMBO, HAVO, and VWO. The Dutch secondary education system uses distinct academic tracks that differ in level and future study pathways. VMBO (Pre‐Vocational Secondary Education) provides 4 years of pre‐vocational education, HAVO (Senior General Secondary Education) offers a 5‐year general secondary program preparing students for universities of applied sciences, and VWO (Pre‐University Education) delivers the highest academic track, providing 6 years of education leading to theoretical universities.

To improve the generalizability of the study, schools were recruited from the Amsterdam region to represent an urban population and from surrounding villages in the province of Utrecht to represent a more rural population. Participation was granted based on school approval, after which the questionnaires were administered to the students.

In addition, the sample included higher education students from HBO (universities of applied sciences) and universities. These students were recruited through channels commonly used by student populations, including study associations, student organizations, and sports clubs. Among them were also medical students and dental students. All participants were provided with informed consent. Participation was voluntary, and responses were processed anonymously. The online questionnaire was distributed by sending the survey link to students. The final sample encompassed a diverse set of target groups. General findings, such as the overall percentage of correct responses to specific questions and self‐perceived knowledge levels, were derived from this comprehensive cohort. This included students from all secondary education backgrounds (VMBO, HAVO, and VWO), as well as higher education students (HBO and university), ensuring a broad representation of educational backgrounds.

### 2.2 Instrument

A 16‐item questionnaire (see Appendix 1: Questionnaire) was developed to assess awareness and sense of urgency regarding the management of dental avulsion. The questionnaire consisted of two parts.

Part I contained questions on demographics and self‐perceived knowledge of dental trauma, and to identify the first point of contact in case of dental trauma.

**FIGURE 1:**
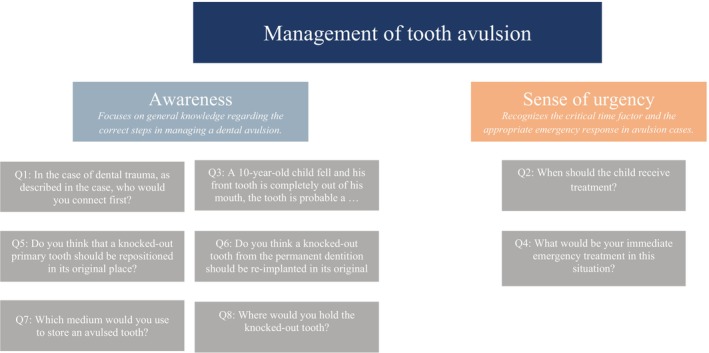
Division of the eight avulsion management questions into two categories: Awareness and sense of urgency.

Part II consisted of eight multiple‐choice questions divided into two domains, designed around a hypothetical case scenario involving dental avulsion (Figure 1).

1. Sense of awareness, reflecting general knowledge on proper management steps.
2. Sense of urgency, referring to recognition of the critical time factor and appropriate emergency response.

The questionnaire was a modified version of those previously used in several studies [14, 15, 16]. Two experts (L.D. and R.H.) validated the questionnaire for content validity. Two students (M.R. and R.R.) evaluated the questionnaire for face validity. After validation, the questionnaire was pilot‐tested with five participants. Small adjustments were made based on their feedback. The final version of the questionnaire was uploaded on Qualtrics (Qualtrics, Provo, UT), an online survey platform.

In addition to calculating total scores, participants were classified into a dichotomous outcome variable. This classification was based on national clinical guidelines for avulsion management and required that participants answer six specific questions correctly (Q2, Q4, Q5, Q6, Q7, Q8) [4]. Only those who answered all six questions correctly were considered theoretically capable of managing a dental avulsion without compromising the prognosis in this study.

### 2.3 Statistical Analysis

For the final statistical analysis, specific inclusion and exclusion criteria were applied to ensure consistency and reliability across predefined groups. Inclusion criteria required informed consent and complete responses to questions assessing awareness, urgency, and guideline‐specific knowledge. Exclusion criteria encompassed individuals with practical medical experience (e.g., medical specialists, dentists), as well as those with a medical background not directly related to medical or dental school education.

To facilitate subgroup analyses, particularly comparisons of educational levels, participants were categorized into the following educational groups: Pre‐Vocational Secondary Education (VMBO), Senior General Secondary Education (HAVO), Pre‐University Education (VWO), Higher Professional Education (HBO), and University programs (further distinguished by medical and non‐medical backgrounds). Participants' responses were imported into SPSS for statistical analysis.

### 2.4 Statistical Analysis

Descriptive statistics were used to summarize demographic variables. One‐way ANOVA was performed to examine differences in mean awareness and urgency scores across educational levels. When the assumption of homogeneity of variances was violated, Games‐Howell post hoc tests were used. All tests used a significance level of *p* < 0.05.

## 3 Results

### 3.1 Participant Characteristics and Descriptive Statistics

A total of 373 participants completed the questionnaire. After excluding those who did not provide informed consent, 348 responses were included in the analysis. Table 1 presents detailed demographic characteristics, while Table 2 presents self‐perceived knowledge and healthcare‐seeking behavior.

**TABLE 1:** Demographic characteristics.

	*N* = 348	%
Gender		
Male	107	31
Female	236	67
Non‐binary	2	1
I prefer not to respond	3	1
Age (years)		
5–14	12	3
15–24	251	72
25–34	31	9
35–44	16	5
45–54	30	9
> 55	8	2
Education		
Primary education	10	3
Highschool		
VMBO (pre‐vocational secondary education)	45	13
HAVO (senior general secondary education)	53	15
VWO (pre‐university education)	60	17
Higher education		
MBO (secondary vocational education)	9	3
HBO (higher professional education)	35	10
WO (university education)	129	37
None	5	1
Did not answer	2	1
Medical background		
No	227	65
Yes	121	35
Currently studying Medicine	58	16
Currently studying Dentistry	29	8
Do you have personal experience with dental trauma?		
No	265	78
Yes	75	22

**TABLE 2:** Participants' self‐assessment of knowledge and methods for seeking healthcare in the case of dental trauma.

Question	*N* = 348	%
Self‐assess your knowledge of dental trauma		
None	88	25
Little	148	43
Average	81	23
High	23	7
Did not answer	8	2
How would you start searching for a healthcare provider in the case of dental trauma?		
Online via Google	64	18
Ask the dentist for advice	187	54
Call 112 (emergency service)	9	3
Ask friends/family	22	6
Via social media	3	1
Ask the GP for advice	20	6
Go directly to the emergency room	22	6
Other	11	3
Did not answer	10	3

The results regarding the management of dental avulsion (see Table 3) highlight critical knowledge gaps with regard to the urgency of treatment. Although 70% of respondents correctly identified a dentist or emergency dentist as the first point of contact (Q1), only half of the participants knew that treatment should occur immediately, within 30 min (Q2). Additionally, 60% correctly identified a knocked‐out front tooth in a 10‐year‐old as a permanent tooth (Q3), and only 62% knew the proper emergency response (reimplantation or storage in a suitable medium) (Q4). Furthermore, 82% correctly stated that primary teeth should not be reimplanted (Q5), while 76% (Q6) recognized that permanent teeth should be. Regarding storage, 57% (Q7) selected appropriate media like saliva, saline, or milk. Lastly, 73% of participants (Q8) correctly indicated that the tooth should be held by the crown. Table 4 presents a heatmap visualization of the percentage of correct responses per question across different groups.

**TABLE 3:** Survey results on dental trauma management, number and percentages of correct responses of all participants.

Question	Correct (*N*)	Correct (%)
Q1. In case of dental trauma, which would be the first place you would contact?	221	70
Q2. When should the child be treated?	154	49
Q3. A 10‐year‐old child has fallen, and their front tooth is completely out of the mouth. The tooth is likely a… (permanent tooth/primary tooth/I don't know):	187	60
Q4. What would your immediate emergency treatment be in this situation?	193	62
Q5. Do you think a knocked‐out tooth from the primary dentition should be re‐implanted in its original position?	256	82
Q6. Do you think a knocked‐out tooth from the permanent dentition should be re‐implanted in its original position?	239	76
Q7. Which medium would you use to store an avulsed tooth?	178	57
Q8. Where would you hold the knocked‐out tooth?	227	73

**TABLE 4:** Survey results showing the percentage of correct answers per question and group regarding dental avulsion management (Q1–Q8).

Group	Q1%	Q2%	Q3%	Q4%	Q5%	Q6%	Q7%	Q8%
VMBO (pre‐vocational secondary education)	73	30	36	54	68	68	52	52
HAVO (senior general secondary education)	71	54	37	51	79	75	35	56
VWO (pre‐university education)	64	48	69	55	86	79	41	74
HBO (higher professional education)	54	58	69	73	62	62	62	69
University education non‐medical	63	49	74	58	79	77	60	74
Medicine	84	40	64	68	92	84	74	90
Dentistry	92	88	96	100	96	96	100	96

*Note:* Categorization: 20%–40% = red, 40%–60% = orange, 60%–80% = yellow, 80%–100% = green.

### 3.2 Awareness and Sense of Urgency in Dental Avulsion Management

To better understand the specific domains of knowledge related to dental avulsion, the eight questions shown in Table 3 were divided into two conceptual categories: awareness and sense of urgency, as seen in Figure 1. Awareness refers to the general knowledge and understanding of how to manage a tooth avulsion correctly. This includes knowledge about the type of tooth (permanent/primary), appropriate storage media, and correct handling of the avulsed tooth (Q1, Q3, Q5, Q6, Q7, and Q8). A sense of urgency focuses on participants' recognition of the critical time factor in avulsion management and the appropriate emergency actions (Q2 and Q4). This division allows for a more nuanced analysis of which aspects of dental trauma knowledge may be lacking among different educational groups. By analysing the mean scores for awareness and urgency separately, it is possible to identify whether deficiencies lie more in general knowledge or in the recognition of urgency. Separate ANOVA tests were conducted to explore whether educational background significantly influenced performance on either of these two categories.

### 3.3 ANOVA of the Mean Score of Awareness Between Educational Levels

The descriptive statistics for awareness scores (range: 0–6 points) regarding the management of dental avulsion are reported in Table 5. The lowest mean score was associated with pre‐vocational secondary students (*M* = 3.5), and the highest mean score was associated with participants with a dental background (*M* = 5.75). To further interpret the clinical relevance of the results, the overall mean awareness score (*M* = 4.18, SD = 1.46) was used as a reference threshold. Educational groups scoring below this average demonstrate lower awareness of dental avulsion management. Based on this reference, four out of seven groups scored below the overall average (VMBO (*M* = 3.5), HAVO (*M* = 3.52), VWO (*M* = 4.12), HBO (*M* = 3.77)). The largest variability in awareness was found in the HBO group (SD = 1.90), suggesting inconsistent knowledge within that group. In contrast, participants with a dental background had the smallest SD (0.44). Only 19% of participants (*n* = 52) answered all six awareness questions correctly (Table 8).

**TABLE 5:** Descriptive statistics for awareness scores on dental avulsion by educational background, referenced to medicine and dentistry groups.

Group	*N*	*M*	SD	95% CI	Sig.	*p*
VMBO (pre‐vocational secondary education)	44	3.50	1.62	[3.01–3.99]	Medicine	< 0.001
					Dentistry	< 0.001
HAVO (senior general secondary education)	50	3.52	1.28	[3.16–3.88]	Medicine	< 0.001
					Dentistry	< 0.001
VWO (pre‐university education)	42	4.12	1.15	[3.76–4.48]	Medicine	0.027
					Dentistry	< 0.001
HBO (higher professional education)	26	3.77	1.90	[3–4.54]	Dentistry	< 0.001
University education non‐medical	42	4.26	1.19	[3.89–4.63]	Dentistry	< 0.001
Medicine	50	4.88	1.08	[4.57–5.19]	VMBO	< 0.001
					HAVO	< 0.001
					VWO	0.027
					Dentistry	< 0.001
Dentistry	24	5.75	0.44	[5.56–5.94]	VMBO	< 0.001
					HAVO	< 0.001
					VWO	< 0.001
					HBO	< 0.001
					University education	< 0.001
					Non‐medical	
					Medicine	< 0.001
Total	278	4.18	1.46	[4.01–4.35]		

To explore whether awareness differed between education levels, we compared the groups using a one‐way ANOVA test. Levene's test was significant (*p* < 0.05), indicating that the assumption of homogeneity of variances was violated. Therefore, the Games‐Howell post hoc test was used. The ANOVA shows statistically significant differences in awareness between education levels, *F* (6.271) = 12.87, *p* < 0.001. The effect size, as indicated by eta‐squared (*η* ^2^ = 0.222), suggests that 22% of the variance in awareness is explained by differences in education level. Post hoc analysis indicates that participants with a dental background scored significantly higher in awareness compared to other groups, including medical students (*p* < 0.05). It is also shown that higher professional education (HBO) and non‐medical university programs do not score significantly higher in awareness compared to secondary school students.

### 3.4 ANOVA of the Mean Score of Sense of Urgency Between Educational Levels

The descriptive statistics for sense of urgency (range: 0–2) are reported in Table 6. The lowest mean score was associated with VMBO (pre‐vocational secondary) students (*M* = 0.84), and the highest mean score was associated with participants with a dental background (*M* = 1.88).

**TABLE 6:** Descriptive statistics for sense of urgency (range: 0–2) regarding the management of dental avulsion, presented by educational level.

Group	*N*	*M*	SD	95% CI	Sig	*p*
VMBO (pre‐vocational secondary education)	43	0.84	0.75	[0.61–1.07]	Dentistry	< 0.001
HAVO (senior general secondary education)	51	1.04	0.70	[0.84–1.23]	Dentistry	< 0.001
VWO (pre‐university education)	42	1.02	0.72	[0.80–1.25]	Dentistry	< 0.001
HBO (higher professional education)	26	1.31	0.79	[0.99–1.63]	Dentistry	0.029
University education non‐medical	43	1.7	0.74	[0.84–1.30]	Dentistry	< 0.001
Medicine	50	1.08	0.70	[0.88–1.28]	Dentistry	< 0.001
Dentistry	24	1.88	0.34	[1.73–2.02]	—	
Total	279	1.11	0.74	[1.03–1.20]		

*Note:* A *p*‐value < 0.05 indicates a statistically significant difference in urgency scores between the group and the Dentistry group.

Abbreviations: 95% CI = 95% confidence interval; *M*, mean score; *N*, number of participants; SD, standard deviation; Sig. (compared to Dentistry), significance level of pairwise comparison with the Dentistry group using Games‐Howell post hoc test (*p*‐value).

To further interpret the clinical relevance of the results, the overall mean sense of urgency score (*M* = 1.11, SD = 0.75) was used as a reference threshold. Educational groups scoring below this average can be considered to demonstrate a relatively lower awareness regarding dental avulsion management. Based on this reference, four out of seven groups scored below the overall average (VMBO (*M* = 0.84), HAVO (*M* = 1.04), VWO (*M* = 1.02), Medicine (*M* = 1.08)). Only 34% of participants (*n* = 94) answered both urgency questions correctly (Table 8).

**FIGURE 2:**
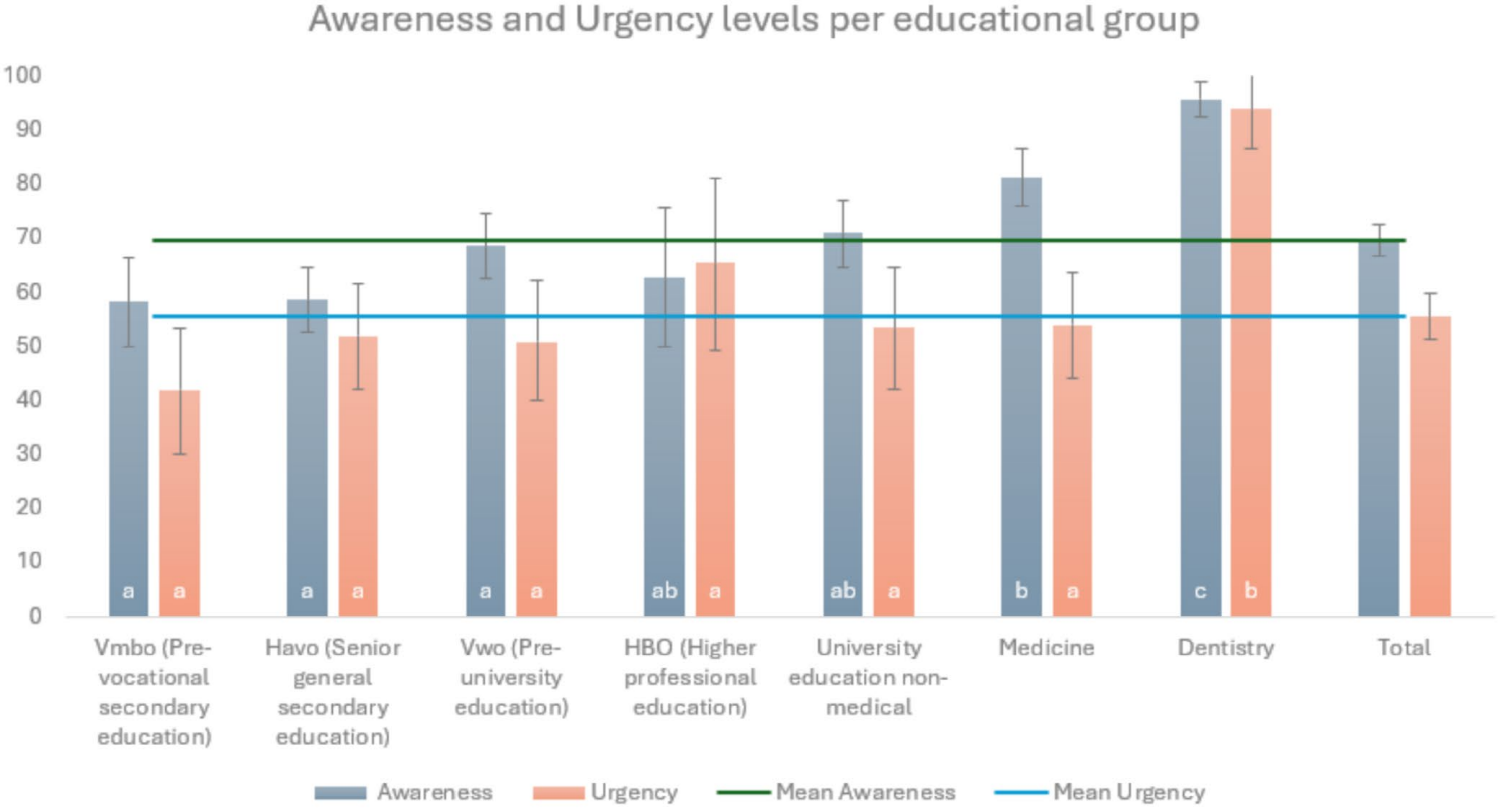
Bar chart with mean awareness and urgency scores per educational group, expressed as percentages (0%–100%). Error bars represent 95% confidence intervals. Horizontal lines indicate the overall mean awareness and urgency scores for the educational groups. Significance letters indicate results from Bonferroni post hoc comparisons (*p* < 0.05). Groups sharing the same letter are not significantly different from each other.

To examine differences in sense of urgency in the management of dental avulsion by education level, an ANOVA was performed. Levene's test was significant (*p* < 0.05), indicating that the assumption of homogeneity of variances was violated. Therefore, the Games‐Howell post hoc test was used. The ANOVA suggested statistically significant variation in awareness among the different education levels, *F* (6.272) = 6.44, *p* < 0.001. The effect size, as indicated by eta‐squared (*η* ^2^ = 0.124), suggests that 12% of the variance in sense of urgency in managing dental avulsion is explained by differences in education level. The post hoc analysis indicates that participants with a dental background exhibit a significantly higher sense of urgency in managing dental avulsion than other groups, including medical students (*p* < 0.05). Interestingly, medical students did not score significantly higher than participants with a secondary education background or those with higher education (HBO or non‐medical university degrees). A visual depiction of the means and the 95% confidence intervals is presented in Figure 2.

Table 7 and Figure 2 present the normalized awareness and urgency scores across educational groups, scaled to a 0%–100% format for ease of interpretation. On average, the total awareness score reached 70%, while the total urgency score was the lowest at 56%. These results highlight that while general awareness is moderate across most groups, urgency remains considerably lower.

**TABLE 7:** Scores for awareness and urgency (normalized to 0%–100%) regarding the management of dental avulsion, presented per educational group.

Group	Awareness % = (*M*/6)*100	Urgency % = (*M*/2)*100
VMBO (pre‐vocational secondary education)	58	42
HAVO (senior general secondary education)	59	52
VWO (pre‐university education)	69	51
HBO (higher professional education)	63	66
University education non‐medical	71	54
Medicine	81	54
Dentistry	96	94
Total	70	56

*Note:* Categorization: 20%–40% = red, 40%–60% = orange, 60%–80% = yellow, 80%–100% = green.

Table 8 presents the number and percentage of participants within each educational group who answered all awareness questions (6 points) or both urgency questions (2 points) correctly.

**TABLE 8:** Number and percentage of participants per educational group who answered all awareness or urgency questions correctly regarding the management of dental avulsion.

Group	Awareness correct *N* (6 points)	Awareness correct % within group	Urgency correct *N* (2 points)	Urgency correct % within group
VMBO (pre‐vocational secondary education)	2	5	9	21
HAVO (senior general secondary education)	2	4	13	26
VWO (pre‐university education)	3	7	11	26
HBO (higher professional education)	5	19	13	50
University education non‐medical	5	12	13	30
Medicine	17	34	14	28
Dentistry	18	75	21	88
Total	52	19	94	34

Across all educational groups, 19% (*n* = 52) of participants achieved a perfect score for awareness, while 34% (*n* = 94) achieved a perfect score for urgency. The lowest percentages of entirely correct responses were found among participants from secondary education levels. For awareness: 5% (VMBO), 4.0% (HAVO), and 7% (VWO). For urgency: 21% (VMBO), 26% (HAVO), and 26% (VWO), respectively. Notably, although medical students demonstrated higher mean scores in awareness and urgency, only 28% of them answered all urgency questions correctly. This is below the overall average of 34%. In contrast, participants with a dental background showed the highest percentage of entirely correct responses for both domains, with 75% scoring full marks on awareness and 87.5% on urgency.

### 3.5 Proportion of Participants Aligning With Clinical Guidelines for Avulsion Management (Awareness+Sense of Urgency)

**FIGURE 3:**
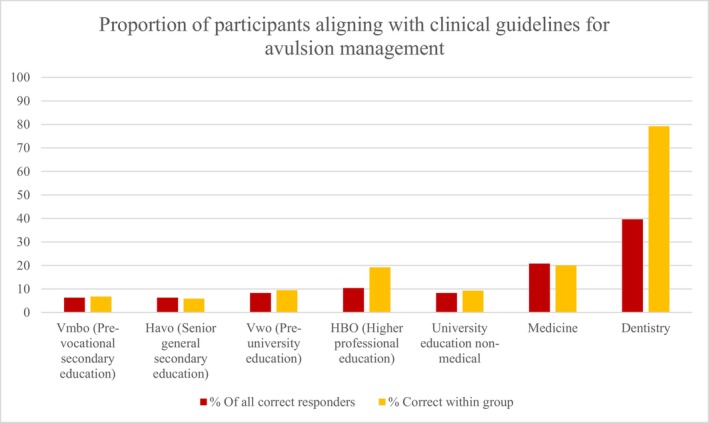
Proportion of participants per educational group who answered all six avulsion management questions correctly in line with clinical guidelines (Q2, Q4–Q8) (*n* = 280). The blue bars represent the percentage of all correct responders (educational groups). The orange bars show the percentage of correct responses within each educational group.

A chi‐square test was conducted to determine whether the proportion of participants who were theoretically capable of managing a dental avulsion in accordance with clinical guidelines without causing harm differed by educational level. The variable indicated whether a participant answered six clinical management questions correctly for tooth avulsion (Q2, Q4–Q8). The correct answers are based on national guidelines for managing a dental avulsion [4]. The chi‐square test showed a significant association between educational level and the likelihood of answering all six questions correctly, *χ* ^2^ (6, *N* = 280) = 76.8, *p* < 0.001. Cramér's *V* = 0.524, *p* < 0.001. This indicates that theoretical competence in avulsion management significantly varied across educational backgrounds. Descriptive statistics revealed that only 17% of the included participants (*N* = 280) provided correct answers to all six questions. Among education groups, participants with a dental background stood out, with 79% answering all questions correctly. Although they represented a smaller portion of the total sample, they accounted for 40% of all participants who provided theoretically correct answers. In contrast, secondary education groups (VMBO, HAVO and VWO) had the lowest percentages of correct responses, ranging from 5.9% to 9.5% within their respective groups. Interestingly, medical students performed only slightly better than the general population, with 20% scoring perfectly. Non‐medical higher education participants (HBO and university non‐medical) also showed differences in knowledge, with 19% and 9%, respectively. Standardized residuals indicated that dentistry students scored significantly above expected values (*z* = 7.3), while secondary education groups scored less than expected (VMBO *z* = −1.7, HAVO *z* = −1.9). These results are summarized in Table 9 and illustrated in Figure 3.

**TABLE 9:** Participants with correct responses to questions Q2, Q4–Q8 per educational group, including standardized residuals.

Group (education level)	*N*	% Correct within group	% Correct to overall correct response rate	Standardized residual
VMBO (pre‐vocational secondary education)	3	7	6	−1.7
HAVO (senior general secondary education)	3	6	6	−1.9
VWO (pre‐university education)	4	10	8	−1.2
HBO (higher professional education)	5	19	10	0.3
University education non‐medical	4	9	8	−1.2
Medicine	10	20	21	0.5
Dentistry	19	79	40	7.3

## 4 Discussion

A critical dimension of effective dental trauma care lies in the immediate recognition of the injury and the swift initiation of appropriate emergency management. Since awareness and a sense of urgency are essential, particularly in cases of dental avulsion, where treatment delay is directly correlated with a poor prognosis [2, 17]. This study therefore assessed these factors among Dutch students, with a focus on knowledge gaps and differences across educational levels. Despite this well‐established urgency, our findings underscore a substantial gap in both awareness and recognition of urgency across the general population, including among those in health‐related fields.

Our study reveals that only 17% of respondents were theoretically capable of managing a dental avulsion in accordance with established guidelines [4]. Most alarmingly, fewer than half (49%) of all participants correctly identified the 30‐min time frame as critical for convenient treatment [18]. This finding aligns with earlier research showing insufficient public knowledge about avulsion management across diverse settings [19, 20, 21]. Particularly concerning is the performance of secondary school students (VMBO, HAVO, and VWO), only 30% of whom recognized the critical treatment window. Given that adolescents and young adults are often present in environments, such as schools and sports facilities, where dental trauma frequently occurs, their low level of awareness poses a significant public health concern. This finding aligns with earlier research indicating that schoolteachers and sports coaches also demonstrate limited knowledge regarding dental trauma and its acute management [19, 20]. The lack of preparedness among these groups, who are often the first responders in dental emergencies, underscores the need for targeted education in these settings.

Equally notable is the performance of medical students, a group presumed to possess a baseline level of healthcare knowledge. In this study, they did not outperform their non‐medical higher education peers in recognising the urgency of dental avulsion management. Only 40% correctly identified the need for immediate intervention—well below the 65% observed among community health workers in Brazil [22]. This discrepancy may be due to 28% of the community health workers having assisted with dental trauma, which has provided them with relevant experience and enhanced their practical knowledge. Nonetheless, a 40% correct rate indicates a gap in current medical curricula regarding emergency dental care and points to a broader issue: the insufficient integration of oral health into general medical education [23, 24]. These findings support prior calls for stronger interdisciplinary collaboration and for incorporating dental trauma management into medical training programs [25, 26].

Self‐assessed knowledge further illustrates the magnitude of the problem: 70% of participants rated their knowledge as ‘none’ or ‘little’. Although most participants indicated they would seek help from a dentist (55%) or use Google (19%), this reactive behavior underscores the lack of preparedness for on‐the‐spot decision‐making in trauma scenarios. The almost negligible reliance on social media (1%) as a source of guidance may reflect mistrust in the clinical reliability of such platforms or a general unfamiliarity with their use for health‐related decision‐making. However, this also presents a valuable opportunity: given the popularity of digital media formats among adolescents and young adults, social media could serve as an effective channel for disseminating evidence‐based information on dental trauma management, particularly when designed in engaging and accessible formats [27].

Despite the strengths of this study, particularly its broad demographic representation across a wide age range and diverse educational backgrounds, several limitations must be acknowledged. First, the reliance on self‐reported data introduces the potential for bias. Participants may have guessed answers or sought information during the questionnaire, leading to an overestimation of actual knowledge levels. Furthermore, prior exposure to dental trauma may have influenced the results. In our study, 22% of respondents reported prior dental injury. This was particularly common among dentistry students, of whom 45% indicated having experienced such trauma, possibly contributing to their higher knowledge scores. Although the sample included older individuals who were no longer engaged in formal education, thereby enhancing the generalizability of overall knowledge levels to the broader population, some subgroups were underrepresented. For instance, participants from vocational education (MBO) and primary education were excluded from the ANOVA analysis due to insufficient numbers, limiting statistical comparisons for this group.

Importantly, this study provides a nuanced overview of both awareness and sense of urgency in managing dental avulsion, two domains that are essential in emergency dental care. By dividing the knowledge questions into these conceptual categories, the study offers a more nuanced understanding of the most critical knowledge gaps. By conceptually distinguishing these domains, we identified specific knowledge gaps, with the most pronounced deficiencies observed among secondary school students (VMBO, HAVO, and VWO). Given the observed knowledge deficits, especially among lower educational levels, it is recommended that information campaigns and educational programs be targeted at these groups. Schools, sports clubs, and other institutions could help spread basic dental first‐aid knowledge among teenagers and adolescents. In addition, incorporating dental first aid education into general first aid courses could increase awareness and urgency around dental avulsion. Previous research has demonstrated the effectiveness of educational tools in enhancing understanding of traumatic dental injuries (TDI). A study by Özveren and Yıldırım [28] evaluated the impact of an animated educational video on children's knowledge regarding TDI management. Their findings revealed a significant improvement in the children's scores after watching the video, highlighting its effectiveness in increasing awareness of how to manage dental injuries [28]. Future studies could focus on developing and evaluating educational videos tailored to specific target groups that may be at risk of dental trauma.

## 5 Conclusion

This study reveals substantial knowledge gaps in awareness and sense of urgency regarding the emergency management of dental avulsion across educational backgrounds. While some groups exhibited a baseline understanding of dental trauma, recognition of the time‐sensitive nature of avulsion management, particularly the need for replantation within 30 min, was consistently lacking among non‐dental participants.

These deficiencies underscore the critical need for targeted educational interventions, with particular attention to secondary school students and professionals outside the dental field who are likely to witness or respond to such injuries. Future educational efforts should prioritize disseminating concise, accessible information that emphasizes the urgency of avulsion care. Additionally, further research is warranted to evaluate the effectiveness of specific educational tools and to identify other contributing factors that may delay appropriate first aid or clinical intervention in cases of dental avulsion.

## Author Contributions

Study design: L.D., R.H. Data collection: H.M.C., J.A. Writing the main text: H.M.C., J.A. Editing and supervising: L.D., R.H.

## Funding

The authors have nothing to report.

## Ethics Statement

This research was approved by the Ethic Committee of Academic Center of Dentistry Amsterdam (ACTA), The Netherlands.

## Conflicts of Interest

The authors declare no conflicts of interest.

## Data Availability

The data that support the findings of this study are available on request from the corresponding author. The data are not publicly available due to privacy or ethical restrictions.

## Appendix 1

**Table:**

Questionnaire		
Part I		
Number	Question	Options
1	What is your gender?	Male Female Non binary I prefer not to answer
2	What is your age group?	5–14 years 15–24 years 25–34 years 35–44 years 45–54 years 55+
3	What is your level of education or the highest degree you have obtained?	None Primary education Pre‐vocational secondary education (VMBO) Senior general secondary education (HAVO) Pre‐university education (VWO) Secondary vocational education (MBO) Higher professional education (HBO) University (WO) Other:
4	Do you have a medical background or are you attending medical education?	Yes No
5	If yes (4), what is your function or study?	
6	Do you have personal experience with dental injury?	Yes No
7	Assess your knowledge of dental trauma	None Little Average A lot
8	How would you start searching for a healthcare provider in case of dental trauma?	Online via Google Via social media Asking friends/family Asking the general practitioner for advice Asking the dentist for advice Going directly to the emergency department Calling emergency services (112) Other:

Part II		
Case: a 10‐year‐old child is playing outside in the afternoon when he trips over a loose paving stone and falls face‐first onto the ground. As a result of the fall, the child has completely lost one of his upper front teeth, which is no longer present in his mouth. Upon examination, visible bleeding is observed from the gum where the tooth was lost. The child is visibly upset and crying.		
1	In the case of dental trauma, as described in the case, who would you contact first?	General practitioner Hospital Dentist^b^ Emergency dentist^b^ Other:
2	When should the child receive treatment?	When the parent or guardian is available to bring the patient for examination and treatment. During the break or after school. After enough rest. When the patient feels relaxed and wants treatment. Immediately (within 30 min)^b^ Within 4 h Within 24 h Within 48 h I don't know Other:
3	A 10‐year‐old child fell and his front tooth is completely out of his mouth, the tooth is probably a …	Permanent tooth.^b^ Primary tooth. I don't know.
4	What would be your immediate emergency treatment in this situation?	Put the tooth back or carry the tooth in a solution to the dentist.^b^ Stop the bleeding and do nothing with the tooth. I don't know
5	Do you think that a knocked‐out primary tooth should be repositioned in its original place?	Yes No^b^ I don't know
6	Do you think that a knocked‐out permanent tooth should be repositioned in its original place?	Yes^b^ No I don't know
7	Which medium would you use to store a knocked‐out tooth?	The tooth is useless, do not spend time finding or treating it Gauze or tissue Cold milk^b^ Physiological saline solution^b^ Patient's saliva^b^ Tap water Distilled water Store dry in a container or plastic bag Disinfectant solution I don't know Other
8	Where would you hold the knocked‐out tooth?	The crown^b^ The root Both, the crown and root I don't know
9	What would hold you back from putting back a tooth yourself?	

^a^ This questionnaire has been translated from Dutch. The questionnaire was administered in Dutch to the participants in this study.

^b^ Correct answers given maximum one point.
